# 1-Chromonyl-5-Imidazolylpentadienone Demonstrates Anti-Cancer Action against TNBC and Exhibits Synergism with Paclitaxel

**DOI:** 10.3390/ijms21165777

**Published:** 2020-08-12

**Authors:** Karan Modi, Scott Lawson, Guanglin Chen, Deepthi Tumuluri, Inga Rekhtman, Michael Kurtz, G. Cristina Brailoiu, Qiao-Hong Chen, Ashakumary Lakshmikuttyamma

**Affiliations:** 1 Department of Pharmaceutical Sciences, Jefferson College of Pharmacy, Thomas Jefferson University, Philadelphia, PA 19107, USA; karan.modi@jefferson.edu (K.M.); scottlawson13@gmail.com (S.L.); Deepthi.Tumuluri@jefferson.edu (D.T.); Inga.Rekhtman@jefferson.edu (I.R.); michael.kurtz@jefferson.edu (M.K.); gabriela.brailoiu@jefferson.edu (G.C.B.); 2 Department of Chemistry, California State University, Fresno, CA 95819, USA; chen.bmc@gmail.com (G.C.); qchen@csufresno.edu (Q.-H.C.)

**Keywords:** curcumin, triple-negative breast cancer, paclitaxel, IL-6

## Abstract

Curcumin has been well studied for its anti-oxidant, anti-inflammatory, and anti-cancer action. Its potential as a therapy is limited due to its low bioavailability and rapid metabolism. To overcome these challenges, investigators are developing curcumin analogs, nanoparticle formulations, and combining curcumin with other compounds or dietary components. In the present study, we used a 1-chromonyl-5-imidazolylpentadienone named KY-20-22 that contains both the pharmacophore of curcumin and 1,4 benzopyrone (chromone) moiety typical for flavonoids, and also included specific moieties to enhance the bioavailability. When we tested the in vitro effect of KY-20-22 in triple-negative breast cancer (TNBC) cell lines, we found that it decreased the cell survival and colony formation of MDA-MB-231 and MDA-MB-468 cells. An increase in mitochondrial reactive oxygen species was also observed in TNBC cells exposed to KY-20-22. Furthermore, KY-20-22 decreased epithelial–mesenchymal formation (EMT) as evidenced by the modulation of the EMT markers E-cadherin and *N*-cadherin. Based on the fact that KY-20-22 regulates interleukin-6, a cytokine involved in chemotherapy resistance, we combined it with paclitaxel and found that it synergistically induced anti-proliferative action in TNBC cells. The results from this study suggested that 1-chromonyl-5-imidazolylpentadienone KY-20-22 exhibited anti-cancer action in MDA-MB-231 and MDA-MB-468 cells. Future studies are required to evaluate the anti-cancer ability and bioavailability of KY-20-22 in the TNBC animal model.

## 1. Introduction

Curcumin (1,7-bis(4-hydroxy-3-methoxyphenyl)-1,6-heptadiene-3,5-dione) is one of the major polyphenols present in turmeric. Traditionally, turmeric has been used in South Asian countries for microbial infections and any kind of inflammation. Recent studies demonstrated the vast action of curcumin as an anti-oxidant, anti-inflammatory, anti-cancer, and anti-microbial agent [1,2,3,4,5], and its anti-proliferative and anti-metastatic action has been established in breast cancer [3,4,5]. Studies have explored the potential use of curcumin in triple-negative breast cancer (TNBC), a subtype of breast cancer that lacks available therapeutic targets, such as the estrogen receptor (ER) and HER2, as well as the progesterone receptor. Chemotherapy is the current standard therapy for TNBC; however, most patients develop resistance towards the treatment, which results in tumor recurrence and cancer metastasis [6]. One of the other characteristics of TNBC is the BRCA1 mutation in the hereditary phenotype and the silencing of wild type BRCA1 in the sporadic phenotype [7]. Studies have suggested that curcumin could re-express BRCA1 expression in TNBC [8]. Curcumin-exposed HCC-38 and UACC-3199 cells expressed BRCA1 gene expression by reducing the DNA promoter methylation level [9]. Curcumin increased wild type BRCA1 protein expression, phosphorylation, and cellular localization, but not those of mutant BRCA1 [10]. Our recent studies have also suggested that curcumin enhances wild type BRCA1 by modulating chromatin histone acetylation [8].

Various signaling pathways have been detected in curcumin’s anti-cancer activity [11,12]. A major challenge facing curcumin therapy is its low bioavailability, which is due to poor absorption, rapid metabolism, and rapid elimination [13]. To overcome these challenges, various strategies have been identified to improve curcumin action in a physiological system, including the development of curcumin analogs, various types of nanoparticle formulations, and the combination of other dietary components with curcumin [13,14,15]. Curcumin exhibited higher anti-cancer action when it was combined with piperine [16]. A study in breast cancer cells identified that curcumin and piperine reduced breast stem cell self-renewal by targeting lipid metabolism, specifically reducing the stearoyl-CoA desaturase enzyme [17]. A variety of studies have found that curcumin increases its anti-cancer action when it is combined with quercetin, a major polyphenol present in fruits and vegetables [18]. It has been recognized widely for its anti-oxidant and anti-inflammatory action, and reports are also available on its anti-cancer action for various cancers [19,20]. Individually, the bioavailability of curcumin and quercetin is significantly low [21,22]. Studies have shown, however, that quercetin enhances the bioavailability of curcumin in cancer cells [23]. Zhang et al. reported that a combined treatment of curcumin and quercetin increased the apoptosis of gastric cancer cells via the mitochondrial pathway [24]. Our recent studies suggested that the combination of curcumin and quercetin synergistically activated BRCA1 expression and inhibited TNBC cell survival compared to individual treatments of curcumin and quercetin [8]. Another study reported the synergistic anti-proliferative action of curcumin and quercetin in various cancer cells [25]. Mutlu Altundag et al. reported that the combined administration of curcumin and quercetin increases the apoptosis of chronic myeloid cells [26]. Ongoing investigations are focusing on different delivery systems of these compounds using nanoparticles [27,28]. For instance, a very recent study by Mansourizadeh et al. showed that apoferritin nanoparticles loaded with quercetin and curcumin (Que-Cur-HoS-Apo NPs) exhibited a synergistic anti-tumor effect in MCF-7 breast cancer cells [29].

Our approach was to use the dienone KY-20-22 (3-((1*E*,4*E*-5-(1-ethyl-1*H*-imidazol-2-yl)-3-oxopenta-1,4-dien-1-yl)-4*H*-chromen-4-one), which contains the pharmacophore of curcumin and 1,4-benzopyrone (chromone) moiety typical for flavonoids including quercetin. This compound possesses a nitrogen-containing heteroaromatic ring, a molecular weight of 320, a Clog P of 1.89, and a tPSA of 59, implying a high probability of its good oral bioavailability. Earlier studies demonstrated that this compound exhibits anti-proliferative action in prostate cancer cells [30]. The five-membered heterocyclic ring in the dienone has been established as the optimal bioisostere of the phenyl ring in curcumin by making significant contributions to the improved potency in prostate cancer cells and superior bioavailability in rats [31]. Our present study concentrated on the in vitro effect of this 1-chromonyl-5-imidazolylpentadienone KY-20-22 in TNBC cell line models. The dienone dose-dependently inhibited the cell survival and growth of TNBC cell lines MDA-MB-231 and MDA-MB-468. Based on findings that a combination of different chemotherapy drugs or the addition of any therapeutic molecule to chemotherapy drugs enhances the anti-cancer action of the chemotherapy, the present study studied the influence of KY-20-22 on the anti-cancer action of paclitaxel. Our results suggested a synergistic action of paclitaxel and KY-20-22 against TNBC cells.

## 2. Results

### 2.1. Anti-Proliferative Action of 1-Chromonyl-5-Imidazolylpentadienone KY-20-22 in TNBC Cell Lines

Our previous study suggested that curcumin and quercetin act synergistically to inhibit the cell growth of TNBC cells compared to individual treatment [8]. The present study used 1-chromonyl-5-imidazolylpentadienone, KY-20-22, which contains the pharmacophore of both curcumin and quercetin. The previous study suggested that this dienone induced an anti-proliferative effect in prostate cancer cells [30]. Further study found that this dienone is more potent and exhibit higher bioavailability compared to curcumin [31]. To determine the effect of 1-chromone-5-imidazolepentadienone KY-20-22 on TNBC cell survival, an MTT assay was carried out using different doses (0–50 µM) of KY-20-22 for different time courses (24, 48, and 72 h) in MDA-MB-231 and MDA-MB-468 cells. The results suggested a dose-dependent inhibition of the cell survival of both cell lines.

**Figure 1:**
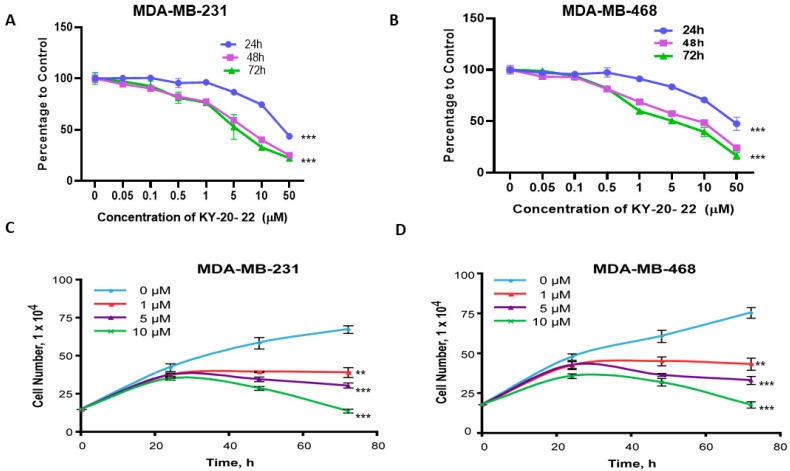

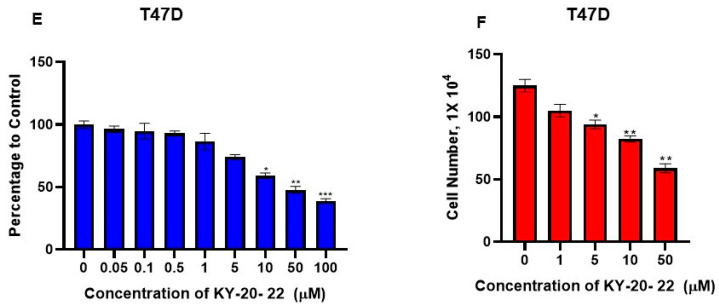
Effect of KY-20-22 on cell survival and growth of triple negative breast cancer TNBC and T47D cell lines. MTT (3-(4,5-Dimethylthiazol-2-yl)-2,5-diphenyltetrazolium bromide) assay of MDA-MB-231 (**A**) and MDA-MB-468 (**B**) cells treated with different concentrations (0–50 µM) of KY-20-22 for different time intervals (0–72 h). Trypan blue exclusion assay of MDA-MB-231 (**C**) and MDA-MB-468 (**D**) cells treated with different concentrations of KY-20-22 (0–50 µM) for 72 h. (**E**) MTT assay and (**F**) trypan blue exclusion assay of T47D cells treated with different concentrations (0–100 µM) of KY-20-22 for 72 h. Each data point represents the mean ± SD from three independent experiments, * *p* < 0.05, ** *p* < 0.01, and *** *p* < 0.001 (vehicle control vs. treatment groups).

After 72 h of treatment, a 10 µM concentration of KY-20-22 reduced the cell survival of MDA-MB-231 by approximately 60% (*p* < 0.001) and that of MDA-MB-468 by approximately 68% (*p* < 0.001) (Figure 1A,B). The IC_50_ value of KY-20-22 was approximately 5 µM for both MDA-MB-231 and MDA-MB-468 cells. Further to determine the effect of KY-20-22 on the growth of MDA-MB-231 and MDA-MB-468 cells, a trypan blue assay was carried out with different concentrations (0–10 µM) of KY-20-22 for different time intervals (24, 48, and 72 h). Vehicle-treated control (0 µM) showed time-dependent growth of both MDA-MB-231 and MDA-MB-468 cells, whereas KY-20-22-treated MDA-MB-231 and MDA-MB-468 cells exhibited a dose-dependent reduction of cell growth, and a significant reduction was observed with a 0.5–1 µM concentration of KY-20-22 (*p* < 0.001, Figure 1C,D). Furthermore, the effect of KY-20-22 was tested in T47D cells, an ER-positive breast cancer line, to test whether the action of KY-20-22 is specific to TNBC cells. We found that KY-20-22 was more effective in TNBC cells compared to T47D. The IC_50_ value of KY-20-22 for T47D was 50 µM. This is 10 times higher than that required for MDA-MB-231 and MDA-MB-468 cells (Figure 1E,F).

**Figure 2:**
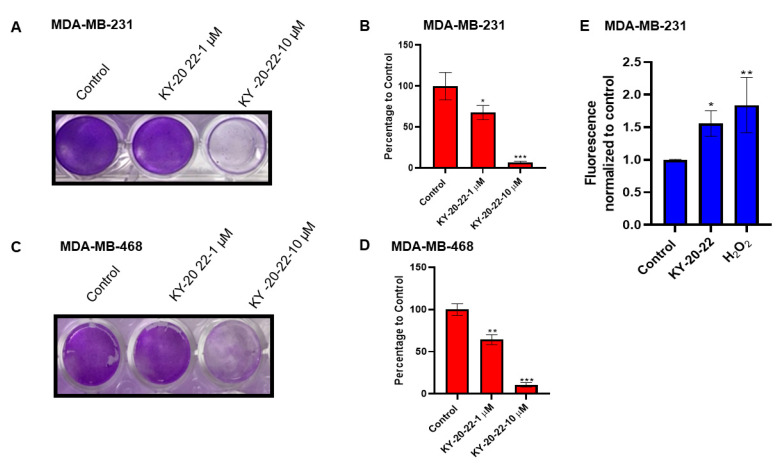
Effect of KY-20-22 on colony formation and mitochondrial ROS generation. Colony formation of (**A**,**B**) MDA-MB-231 and (**C**,**D**) MDA-MB-468 cells treated with different concentrations of KY-20-22 (0–50 µM) for different time intervals (0–72 h). After the treatment period, cells were washed and allowed to grow in normal growth medium for 14 days and then stained with crystal violet. Histograms represent the number of colonies counted using an inverted microscope. (**E**) MDA-MB-231 cells treated with a 25 µM concentration of KY-20-22. H_2_O_2_-treated cells were used as a positive control. Mitochondrial ROS generation was measured as mentioned in the materials and methods. Each data point represents the mean ± SD from three independent experiments, * *p* < 0.05, ** *p* < 0.01, and *** *p* < 0.001 (vehicle control vs. treatment groups).

The inhibition of cell growth by KY-20-22 was further confirmed by a colony formation assay. The results revealed a dose-dependent response, where even a 1 µM concentration of KY-20-22 decreased (*p* < 0.05) the colony formation, and treatment with a 10 µM concentration of KY-20-22 had a significant effect in reducing the colony formation of both MDA-MB-231 and MDA-MB-468 cells compared to vehicle-treated cells (*p* < 0.001) (Figure 2A–D). These studies indicate that KY-20-22 treatment significantly reduced TNBC cell line growth.

Furthermore, we carried out experiments to assess whether KY-20-22 treatment increases ROS generation in MDA-MB-231 cells. Mitochondrial ROS generation was determined in real time using fluorescent MitoSox Red superoxide indicator in MDA-MB-231 cells treated with KY-20-22. The results suggested that KY-20-22 significantly increased mitochondrial ROS generation (Figure 2E). For a positive control, mitochondrial ROS generation was detected in cells treated with hydrogen peroxide. This result suggests that KY-20-22 may induce cell death by generating mitochondrial ROS.

### 2.2. Effect of KY-20-22 on Normal Human Mammary Epithelial Cells

**Figure 3:**
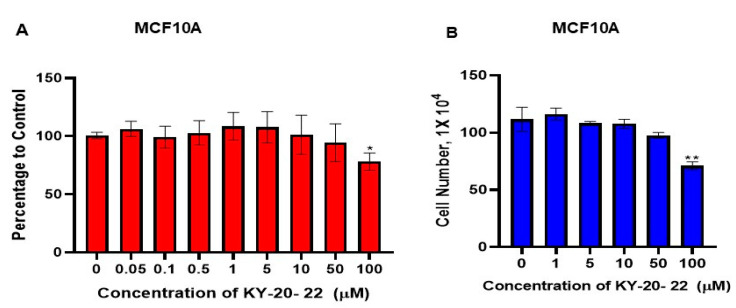
Effect of KY-20-22 on cell survival and growth of MCF10A cells. (**A**) MTT assay of MCF10A cells treated with different concentrations (0–100 µM) of KY-20-22 for 72 h. (**B**) Trypan blue exclusion assay of MCF10A cells treated with different concentrations of KY-20-22 (0–50 µM) for 72 h. Each data point represents the mean ± SD from three independent experiments, * *p* < 0.05, and ** *p* < 0.01 (vehicle control vs. treatment groups).

To assess the toxicity of KY-20-22 on non-cancerous cells, the cell survival of MCF10A, a normal human mammary epithelial cell, was used for further studies. MCF10A was treated with different concentrations of KY-20-22. The result showed that a dose of up to 50 µM of KY-20-22 did not inhibit the survival of MCF10A cells. A significant inhibition of MCF10A cell survival (28% inhibition, *p* < 0.05) was observed with 100 µM of KY-20-22 (Figure 3A). Similarly, the growth of MCF-10A was not significantly decreased by KY-20-22 at lower concentrations (0–50 µM), however, a significant decrease was observed at a 100 µM concentration (Figure 3B). The IC_50_ of KY-20-22 required for TNBC cell inhibition was 5 µM and this concentration did not induce cytotoxicity in MCF10A cells. This result indicates that a lower dose of KY-20-22 is relatively safe in normal breast epithelial cells.

### 2.3. 1-Chromonyl-5-Imidazolylpentadienone KY-20-22 Regulates Genes Involved in Tumor Migration and Inhibits the Migration of TNBC Cell Lines

**Figure 4:**
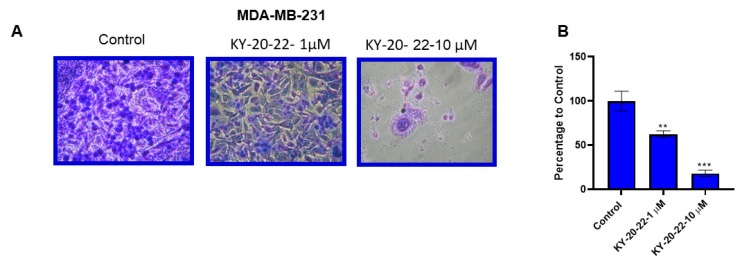
Effect of KY-20-22 on cell migration. (**A**) Boyden chamber assay of MDA-MB-231 cells treated with KY-20-22 (1 and 10 µM), for 24 h. (**B**) Histogram represents the number of cells migrated through the porous membrane in different treatment conditions. Each data point represents the mean ± SD from three independent experiments, * *p* < 0.05, ** *p* < 0.01, and *** *p* < 0.001 (vehicle control vs. treatment groups).

Our previous studies demonstrated that the combination of curcumin and quercetin significantly reduced the migration of TNBC cell lines compared to individual treatments of curcumin and quercetin [8]. In the present study, a Boyden chamber assay was carried out to investigate the effect of 1-chromone-5-imidazolepentadienone KY-20-22 on the migration of TNBC cell lines. We found that treatment with KY-20-22 dose-dependently reduced the migration of MDA-MB-231 cells. The group treated with 10 µM of KY-20-22 exhibited an 80% reduction in the migratory cells compared to the vehicle control (*p* < 0.001) (Figure 4A,B).

**Figure 5:**
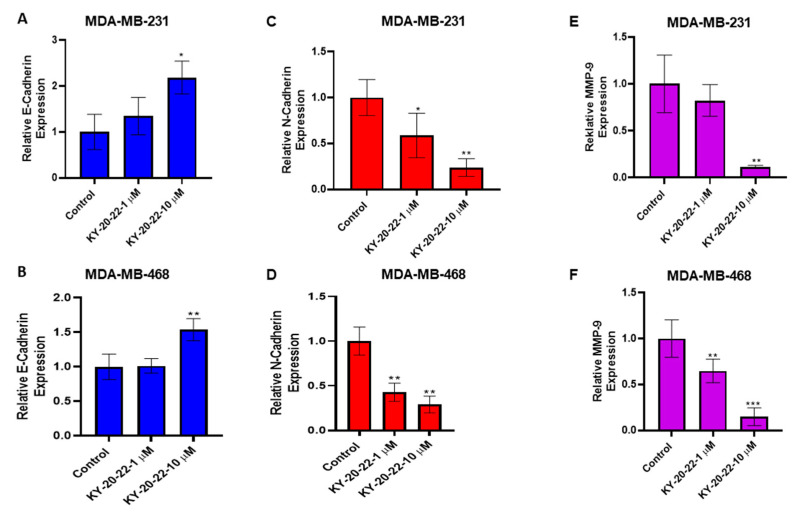
Effect of KY-20-22 on epithelial-mesenchymal transition [EMT] markers. The mRNA expression levels of E-cadherin (**A**,**B**), N-cadherin (**C**,**D**), and matrix metalloproteinase-9 [MMP-9] (**E**,**F**) were analyzed in MDA-MB-231 cells treated with KY-20-22 for 48 h. E-cadherin and N-cadherin expression levels were normalized to GAPDH expression. Each data point represents the mean ± SD from three independent experiments, * *p* < 0.05, ** *p* < 0.01, and *** *p* < 0.001 (vehicle control vs. treatment groups).

To further investigate the action of KY-20-22 on TNBC tumor migration, we studied epithelial–mesenchymal transcription (EMT), which is associated with TNBC progression and migration. Studies were carried out to analyze whether the expression levels of EMT biomarkers may be regulated by KY-20-22 treatment. E-cadherin is one of the biomarkers for epithelial phenotype, and the expression level of E-cadherin is low in mesenchymal cells. Treatment with KY-20-22 (10 µM) significantly increased the E-cadherin expression level for both MDA-MB-231 and MDA-MB-468 cells (two-fold, *p* < 0.05) (Figure 5A,B). A decrease in E-cadherin levels associated with an increase in *N*-cadherin, called “cadherin switching”, is a feature of EMT in cancer metastasis [32,33]. The present study detected a dose-dependent decrease in *N*-cadherin expression with the treatment of KY-20-22, with an approximately five-fold decrease (*p* < 0.01) with 10 µM treatment (Figure 5C,D). Another protein that plays a significant role in cancer cell migration is matrix metalloprotease 9 (MMP-9), the higher expression of which correlates with breast cancer metastasis. Our study determined that treatment with KY-20-22 (10 µM) significantly reduced the expression levels of MMP-9 in both MDA-MB-231 and MDA-MB-468 cells (*p* < 0.001) (Figure 5E,F). All of these results demonstrate that KY-20-22 significantly reduces MDA-MB-231 migration and regulates the signaling molecules involved in EMT and cancer metastasis.

### 2.4. 1-Chromonyl-5-Imidazolylpentadienone KY-20-22 Downregulates Interleukin-6 (IL-6) Expression

**Figure 6:**
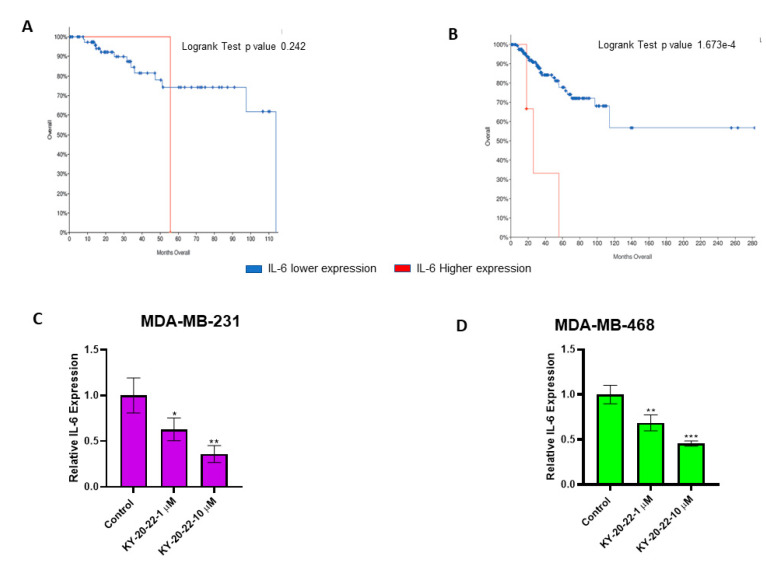
Overall survival rate of TNBC and estrogen receptor negative (ER^−^ ) patients with higher expression of IL-6 and the effect of KY-20-22 on IL-6 expression. Data from the TCGA database containing different breast cancer subtypes, such as TNBC and ER^−^, were extracted and used to generate an overall survival curve using the cBio Cancer Genomics Portal [36,37]. (**A**) TNBC and (**B**) ER^−^ patient survival rate with a high expression of IL-6. The mRNA expression level of IL-6 in MDA-MB-231 (**C**) and MDA-MB-468 (**D**) cells treated with KY-20-22 for 48 h. IL-6 expression was normalized to GAPDH expression. Each data point represents the mean ± SD from three independent experiments, * *p* < 0.05, ** *p* < 0.01, and *** *p* < 0.001 (vehicle control vs. treatment groups).

The present study demonstrated that 1-chromonyl-5-imidazolylpentadienone KY-20-22 inhibits the proliferation and migration of TNBC cell lines. Moreover, our experiments suggested that KY-20-22 increases mitochondrial ROS levels. To further understand the effect of this compound against TNBC, we carried out studies on the action of KY-20-22 on specific cytokines that cause chemotherapy resistance in TNBC. IL-6 is one of the signaling molecules that contributes to chemotherapy resistance in breast cancer [34]. Hartman et al. reported that IL-6 is a key signaling molecule involved in TNBC tumor growth and is also associated with poor overall patient survival [35]. Furthermore, our search using The Cancer Genome Atlas (TCGA) database [36,37] confirmed that IL-6 expression influences the survival of TNBC and ER-negative patients (Figure 6A,B). Specifically, survival was shorter among patients with higher IL-6 expression compared to patients with lower IL-6 expression. Thus, the expression levels of IL-6 were analyzed in MDA-MB-231 and MDA-MB-468 cells after being exposed to KY-20-22. The results suggested that the expression of IL-6 was dose-dependently decreased with KY-20-22 treatment (Figure 6C,D). An approximately three-fold decrease was observed with 10 µM of KY-20-22 in MDA-MB-231 cells (*p* < 0.01), and a two-fold decrease in MDA-MB-468 cells (*p* < 0.001).

### 2.5. Synergistic Action of KY-20-22 and Paclitaxel Against TNBC

**Figure 7:**
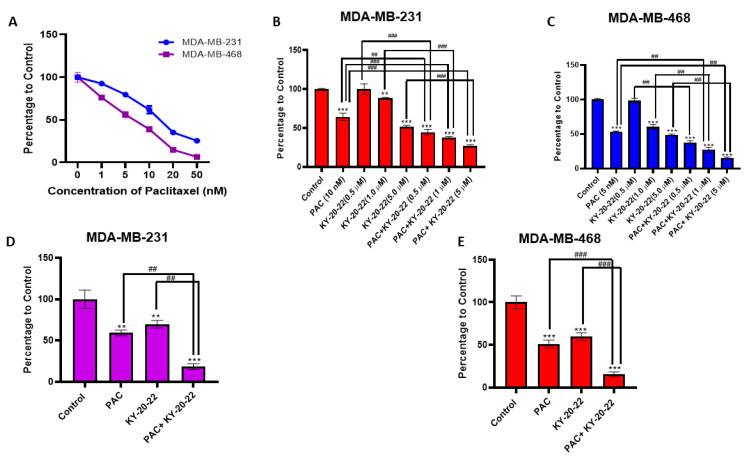
Effect of the combination of paclitaxel and KY-20-22 on cell survival. MTT assay of (**A**) MDA-MB-231 and MDA-MB-468 cells treated with different concentrations of paclitaxel (0–50 nM) for 72 h. MTT assay of (**B**) MDA-MB-231 and (**C**) MDA-MB-468 cells treated with different concentrations of paclitaxel (MDA-MB-231 5 and 10 nM, MDA-MB-468 1 and 5 nM) and KY-20-22 (0.5, 1, and 5 µM) for 72 h. Colony formation of (**D**) MDA-MB-231 and (**E**) MDA-MB-468 cells treated with different concentrations of paclitaxel and KY-20-22 for 72 h. After the treatment period, cells were washed and allowed to grow in normal growth medium for 14 days and then stained with crystal violet. Histograms represent the number of colonies counted using an inverted microscope. Each data point represents the mean ± SD from three independent experiments, ** *p* < 0.01, and *** *p* < 0.001 (vehicle control vs. treatment groups), ## *p* < 0.01, ### *p* < 0.001 (paclitaxel vs. paclitaxel + KY-20-22 or KY-20-22 vs. paclitaxel + KY-20-22).

It was found that 1-chromonyl-5-imidazolylpentadienone KY-20-22 significantly decreased TNBC growth and survival. Furthermore, to identify whether KY-20-22 increases the anti-cancer action of a chemotherapy drug, we studied the combined action of paclitaxel and KY-20-22 in TNBC cell lines. Initially, we studied the dose-dependent effect of paclitaxel on the survival of MDA-MB-468 and MDA-MB-231 cells (Figure 7A). Then, cell survival analysis was carried out using a combination of paclitaxel (5 and 10 nM for MDA-MB-231 and 1 and 5 nM for MDA-MB-468) and different concentrations of KY-20-22 (0.5, 1, and 5 µM). CompuSyn software was used to analyze the combination index (CI) of the combined action of KY-20-22 and paclitaxel. The CI of inhibition of cell survival was below one (CI < 1) with a combination of KY-20-22 (1 µM and 5 µM) and paclitaxel (MDA-MB-231 5 and 10 nM, MDA-MB-468 1 and 5 nM) (Table 1). This result indicates that KY-20-22 and paclitaxel act synergistically to inhibit the survival of TNBC cells. Figure 7B,C represent the combined action of paclitaxel (10 nM for MDA-MB-231 and 5 nM for MDA-MB-468) and KY-20-22 (0.5, 1, 5 µM), with results that indicate that the survival of paclitaxel- and KY-20-22-treated cells was significantly lower compared to individual treatment with either KY-20-22 or paclitaxel.

A cell survival (MTT) assay was carried out for MDA-MB-231 and MDA-MB-468 cells. Cells were treated with different concentrations of KY-20-22 and paclitaxel individually and in combination, as indicated in Table 1, for 72 h. CompuSyn software was used to calculate the combination index (CI) of the combined effect. The synergistic action of KY-20-22 and paclitaxel was determined based on the CI theorem (additive effect (CI = 1), synergism (CI < 1), and antagonism (CI > 1) in drug combinations).

**Figure 8:**
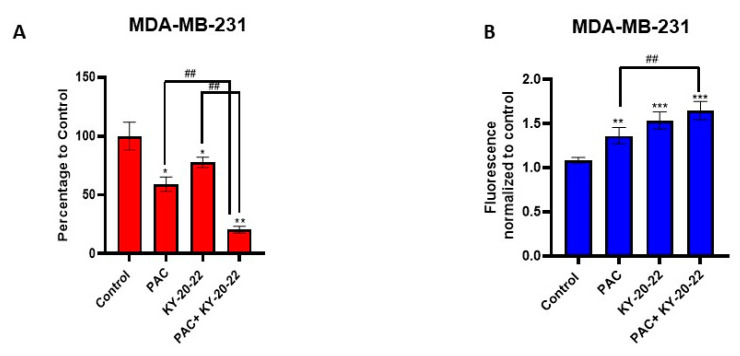
Effect of the combination of paclitaxel and KY-20-22 on cell migration and mitochondrial ROS generation. (**A**) Boyden chamber assay of MDA-MB-231 cells treated with paclitaxel and KY-20-22 for 24 h. Histograms represent the number of cells that migrated through the porous membrane in different treatment conditions. (**B**) MDA-MB-231 cells were treated with paclitaxel (10 µM) and KY-20-22 (25 µM) and mitochondrial ROS generation was measured for 10 min as mentioned in the Materials and Methods (the ROS study used a much higher concentration of drugs than the other studies because ROS generation was measured within 10 min of drug response). * *p* < 0.05, ** *p* < 0.01, and *** *p* < 0.001, ## *p* < 0.01, (paclitaxel vs. paclitaxel + KY-20-22 or KY-20-22 vs. paclitaxel + KY-20-22).

Furthermore, colony formation analysis was carried out to assess the combined action of KY-20-22 (1 µM) and paclitaxel (10 nM for MDA-MB 231 and 5 nM for MDA-MB-468) on the growth of MDA-MB-231 and MDA-MB-468 cells, which revealed that the colonies formed with the combined treatment were significantly reduced with the individual treatment for both cell lines (Figure 7D,E) (*p* < 0.001). These results suggest that KY-20-22 significantly enhances the anti-proliferative action of paclitaxel. Furthermore, a Boyden chamber assay, carried out to assess whether KY-20-22 enhances the anti-migratory action of paclitaxel, revealed that the migration of MDA-MB-231 was significantly (*p* < 0.01) inhibited by the combination of KY-20-22 and paclitaxel treatment compared to individual treatment (Figure 8A).

### 2.6. Combined Action of KY-20-22 and Paclitaxel on Reactive Oxygen Species Generation

We found that a combination of KY-20-22 and paclitaxel synergistically inhibited the survival and colony formation of MDA-MB-231 and MDA-MB-468 cells, but studies also suggested that KY-20-22 alone induced ROS generation in MDA-MB-231 cells. To learn more, we carried out further experiments to determine the effect of the combination of paclitaxel and KY-20-22 on ROS generation in MDA-MB-231 cells. Our findings suggest that the ROS generation was significantly higher in the paclitaxel and KY-20-22 cells compared to individual treatment with paclitaxel, however, there was no significant change when compared to KY-20-22 alone (Figure 8B). Further in vivo studies are required to confirm the combined action of paclitaxel and KY-20-22 against TNBC.

## 3. Discussion

Most chemotherapeutics induce cell cycle arrest and cell death by generating ROS, hence ROS exhibits tumor-suppressing action in different cancer cells [38]. In the present study, we detected that a 1-chromonyl-5-imidazolylpentadienone dose-dependently generated ROS and inhibited the growth and survival of the TNBC cell lines MDA-MB-231 and MDA-MB-468 (Figure 1A–D and Figure 2E). Furthermore, the 1-chromonyl-5-imidazolylpentadienone named KY-20-22 regulated “cadherin switching”, increasing E-cadherin expression and decreasing *N*-cadherin expression in MDA-MB-231 cells (Figure 5A–D). These results suggested that the 1-chromonyl-5-imidazolylpentadienone induced anti-cancer action in TNBC cell lines. Moreover, when we combined KY-20-22 with paclitaxel, the combination synergistically decreased the growth of MDA-MB-231 and MDA-MB-468 cells (Figure 7B–E).

Traditionally, curcumin is used as an anti-inflammatory agent in various parts of the world, and recently it has been identified as possessing anti-cancer activity. Combination therapy approaches, such as the co-administration of curcumin with other dietary components, have shown further benefit in inhibiting cancer growth and metastasis [16,17,18]. Combining curcumin with specific dietary molecules not only increased anti-cancer action but also improved the absorption and bioavailability of curcumin. For instance, the administration of curcumin with piperine, a compound in black pepper, increases the anticancer action of curcumin against colorectal cancer [16] and reduces cancer stem cells in breast cancer [17]. The combined anti-cancer action of curcumin and quercetin has been reported by us and other investigators [8,24,25,29]. In the present study, we found that the 1-chromonyl-5-imidazolylpentadienone KY-20-22 inhibited the survival and proliferation of TNBC cells dose-dependently, with an IC_50_ value of approximately 5 µM (Figure 1A–D). Various reports indicated that the anti-cancer action of curcumin was mediated through increased ROS generation [5]. The findings indicate that the 1-chromonyl-5-imidazolylpentadienone KY-20-22 carries out the biological action of both curcumin and quercetin in generating the anti-proliferative actions and ROS generation. Alterations in the PI3K/AKT/mTOR pathway are common in TNBC [39]. Moreover, there are reports indicating that the individual action of curcumin and quercetin could regulate the PI3K/Akt/mTOR signaling pathways [40,41]. Hence, the anti-proliferative action of KY-20-22 may be due to the regulation of this pathway. It has been found that mTOR signaling is not only deregulated in cancer but also in different pathological conditions and the mTOR pathway is critical to the pathogenesis of HIV-related malignancies [42] and different autoimmune diseases [43]. Hence, KY-20-22 might also be effective in treating different pathological conditions which exhibit deregulated PI3K/Akt/mTOR signaling. Furthermore, 1-chromonyl-5-imidazolylpentadienone KY-20-22 (10 µM) significantly (*p* < 0.01) increased E-cadherin expression and decreased N-cadherin levels in both MDA-MB-231 and MDA-MB-468 cells (Figure 5A–D). These results indicated that KY-20-22 is able to induce “cadherin switching” in TNBC cells. TNBC, a very aggressive breast cancer compared to other subtypes, is a heterogeneous cancer with basal-like and claudin-low TNBC being the major subtypes. Notably, only a portion of basal-like breast cancers are TNBC (77%) [44]. Studies have suggested that basal-like TNBC is associated with mesenchymal features and with a higher expression of EMT genes and a lower expression of E-cadherin or E-cadherin loss [32,44,45]. While our study revealed that the KY-20-22 molecule could modulate the EMT process and alter the mesenchymal features of TNBC, further in vivo studies are required to confirm this.

We found that KY-20-22 significantly reduced MMP-9 expression in MDA-MB-231 cells (Figure 5E,F). MMP-9 expression is regulated by various cytokines and growth factors, with the cytokine IL-6 regulating MMP-9 in macrophage cells [46]. Higher expression levels of IL-6 and IL-8 contribute to TNBC proliferation and metastasis [47] and, moreover, a higher expression of IL-6 is associated with chemotherapy resistance in breast cancer [47,48]. Rincon et al. reported that the response to paclitaxel is lower in breast cancer patients with a higher expression level of IL-6 [48]. In the present study, we used TCGA analysis, which is routinely used to identify novel therapeutic targets in different types of cancers [36,37]. We detected that higher IL-6 expression reduces the survival rate of TNBC and ER-negative patients (Figure 6A,B). All of this information indicates that IL-6 plays a significant role in the pathology of TNBC. We observed significantly lower levels of IL-6 expression in KY-20-22-treated TNBC cells (Figure 6C,D). One of the major events associated with IL-6 binding to its receptor is the activation of signal transducers and activators of transcription-3 (STAT-3). Various studies have indicated that STAT-3 is a molecular target for curcumin via IL-6 modulation for various cancer types [3]. Hence, further studies are required to confirm the action of KY-20-22 in inhibiting the signaling of STAT-3 in TNBC cells. Moreover, IL-6 is a major cytokine involved in different autoimmune diseases. Hence, KY-20-22 might have therapeutic action towards autoimmune diseases such as rheumatoid arthritis, which is treated with the anti-IL-6 receptor tocilizumab [49].

Additionally, we detected that the combination of paclitaxel and KY-20-22 induced a synergistic anti-cancer action against TNBC. Our studies suggested that low concentrations of paclitaxel (5–10 nM) and 1 µM of KY-20-22 inhibited 60–70% of MDA-MB-231 and MDA-MB-468 cell survival (Figure 7B,C). Moreover, the migration of MDA-MB-231 cells was significantly reduced by the combination of KY-20-22 and paclitaxel compared to individual treatment (Figure 8A). Furthermore, various studies reported that curcumin and its analogs increase sensitivity to chemotherapeutic drugs by reversing the multidrug resistance of cancer cells [50,51]. The combined action of paclitaxel and KY-20-22 on TNBC cells may be due to the action of KY-20-22 on multidrug resistance proteins. The induction of cancer cell apoptosis by various chemotherapeutic drugs is associated with cellular ROS generation [38]. The level of ROS generation varies for different drugs, and cellular ROS production is higher for doxorubicin, epirubicin, and daunorubicin and lower for taxanes, vinca alkaloids, and nucleotide analogs [52]. A decrease in cellular anti-oxidant levels and mitochondrial ROS generation are the reasons behind chemotherapy-induced cellular levels in ROS. Hence, it is worthwhile to identify compounds that target ROS levels to treat solid tumors [53]. In this study, we found that the combination of paclitaxel and KY-20-22 significantly increased ROS generation compared to paclitaxel treatment alone in MDA-MB-231 cells (Figure 8B). Therefore, it is possible that the combination of KY-20-22 with paclitaxel increases the anti-cancer activity of paclitaxel or reduces paclitaxel resistance in TNBC. However, further in vivo studies are required to confirm the combined action of paclitaxel and KY-20-22 in the TNBC animal model. Moreover, our study found that KY-20-22 is not toxic to normal mammary cells at the dose required for TNBC cell inhibition. Future animal studies are essential for the detection of the therapeutic index of KY-20-22.

Studies on the pharmacokinetics of KY-20-22, including its absorption, bioavailability, and metabolism, are also needed to confirm the novelty of this compound. In particular, the bioavailability of curcumin/quercetin has been considered a major limitation to achieving their biological activity in the in vivo system [13]. The preparation of various curcumin/quercetin analogs and different formulations have been explored to increase the bioavailability of these compounds [14]. The 1-chromonyl-5-imidazolylpentadienone KY-20-22 is prepared by inserting specific moieties to overcome this limitation. However, our present study did not evaluate the bioavailability of KY-20-22 in any system.

In conclusion, the findings of our present study suggest that the 1-chromonyl-5-imidazolylpentadienone KY-20-22 induced an anti-proliferative effect and enhanced ROS generation in TNBC cells. Furthermore, KY-20-22 regulated different EMT markers and decreased the expression of signaling molecules such as MMP-9 and IL-6. A combination of KY-20-22 and paclitaxel demonstrated a synergistic effect on TNBC growth reduction. These results indicate that KY-20-22 is a novel molecule that encompasses the biological action of both curcumin and quercetin in inducing anti-cancer action in TNBC. Further in vivo studies are required to evaluate the TNBC tumor-reducing efficacy and bioavailability of KY-20-22.

## 4. Materials and Methods

### 4.1. Materials

First, 3-(4,5-Dimethylthiazol-2-yl)-2,5-diphenyltetrazolium bromide (MTT) was purchased from Sigma-Aldrich (St. Louis, MO, USA). MDA-MB-231, MDA-MB-468, and T47D breast cancer cell lines were purchased from ATCC (Manassas, VA, USA). An RNA isolation kit was purchased from Qiagen (Germantown, MD, USA). Taqman gene expression assays and master mix for quantitative polymerase chain reaction (PCR) were purchased from Life Technologies (Waltham, MA, USA).

### 4.2. 1-Chromonyl-5-Imidazolylpentadienone Synthesis

**Figure 9:**
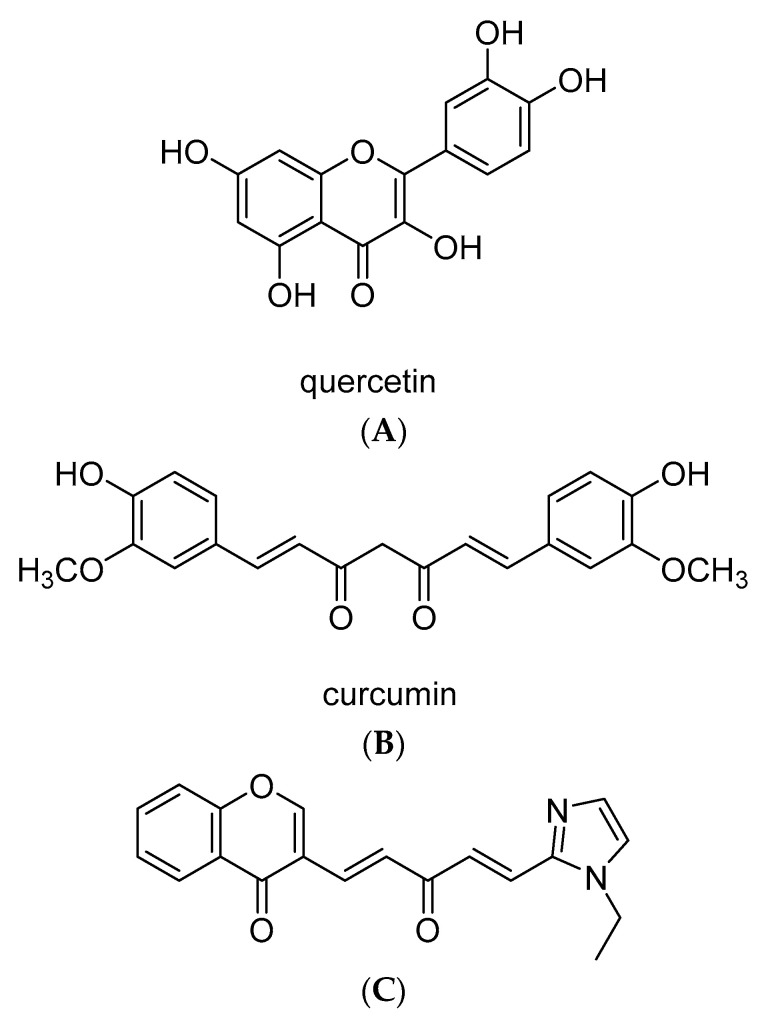
Structure of (**A**) quercetin (**B**) curcumin and (**C**) KY-20-22 (chemical name: 3-((1*E*,4*E*-5-(1-ethyl-1*H*-imidazol-2-yl)-3-oxopenta-1,4-dien-1-yl)-4*H*-chromen-4-one, chemical formula: C_19_H_16_N_2_O_3_, exact mass: 320.1161).

The original design of 3-((1*E*,4*E*-5-(1-ethyl-1*H*-imidazol-2-yl)-3-oxopenta-1,4-dien-1-yl)-4*H*-chromen-4-one was driven by the notion that appreciable synergistic effects can be achieved by integrating two or more privileged pharmacophores into one single hybrid compound [54]. The pharmacophores used were (1*E*,4*E*)-1,5-diaryl-1,4-penta-dien-3-one and chromone from curcumin and quercetin, respectively (Figure 9). The hybrid compound KY-20-22 was prepared via an aldol condensation of 3-formylchromone with (*E*)-4-(1-ethyl-1*H*-imidazol-2-yl) but-3-en-2-one according to the procedure reported earlier [30]. Its ^1^H NMR and ^13^C NMR data are consistent with those reported in the literature [54].

### 4.3. Cell Culture

TNBC cell lines (MDA-MB-231 and MDA-MB-468), and ER-positive (T47D) cells were grown in RPMI medium containing 1% penicillin–streptomycin and 10% fetal bovine serum (FBS) at 37 °C in 5% CO_2_. MCF10A (normal human mammary epithelial) cells were grown in DMEM/F12 medium containing horse serum (5%), EGF (20 ng/mL), hydrocortisone (0.5 mg/mL), cholera toxin (100 ng/mL), insulin (10 µg/mL), and 1% penicillin–streptomycin.

### 4.4. Cell Growth and Survival Assays

The cell survival of KY-20-22 was determined in MDA-MB-231, MDA-MB-468, T47D, and MCF10A cells. Initially, the cells (5 × 10^3^ cells/well) were plated in 96-well plates for 24 h. The attached cells were treated with different concentrations of KY-20-22 (0–50 µM) for 72 h. Control cells were treated with vehicle (low concentration of DMSO that is present in KY-20-22). Cells were treated with 10 µL MTT (5 mg/mL) for 4 h after the treatment period. The plate was incubated for overnight after adding a solubilization reagent to dissolve the purple precipitate obtained from MTT. The absorbance at 570 nm and 650 nm was measured using a microplate reader. The cell growth of KY-20-22-treated MDA-MB-231, MDA-MB-468, T47D, and MCF10A cells was analyzed using trypan blue staining. The different concentrations of KY-20-22 used in these experiments are mentioned in respective figure legends.

### 4.5. Colony Formation Assay

MDA-MB-231 and MDA-MB-468 cells were plated at a density of 0.2 × 10^6^ cells in 24-well plates and treated with KY-20-22 as indicated in the figure legends. The cells treated with vehicle and KY-20-22 were allowed to grow for 2 weeks to form colonies. The growth medium was replenished in a 72 h time interval in all the samples. After 2 weeks, the cells were washed with PBS, fixed with methanol, and stained with crystal violet (0.5% *w*/*v*). The images of the stained cells were taken using an inverted microscope and the colonies were counted.

### 4.6. Boyden Chamber Assay

MDA-MB-231 and MDA-MB-468 cells (2.5 × 10^3^) were exposed to KY-20-22 (1 µM and 10 µM). After 24 h, cells were seeded in Boyden chambers containing matrigel (BioCoat, Franklin Lakes, NJ, USA) and chambers were placed in a 24-well culture dish containing complete growth medium supplemented with chemo-attractant. After 24 h, the migrated/invaded cells on the lower surface of the membrane were stained with crystal violet and counted.

### 4.7. Quantitative RT-PCR Analysis

Isolated total RNA from KY-20-22-treated MDA-MB-231 and MDA-MB-468 cells were reverse transcribed using a Verzo cDNA kit (ThermoFisher Scientific, Waltham, MA). Quantitative RT-PCR of E-cadherin (assay ID: Hs 01023895_m1), *N*-cadherin (assay ID: Hs 00983056_m1), MMP-9 (assay ID: Hs 00234579_m1), and IL-6 (assay ID: Hs 00985641_m1) were carried out using TaqMan gene expression assay method (Life Technologies, Waltham, MA, USA). GAPDH (assay ID: HS02758991_g1) expression was used to normalize mRNA expression levels.

### 4.8. Detection of Mitochondrial ROS Accumulation

The measurement of mitochondrial ROS levels was carried out using the MitoSOX Red superoxide indicator (Molecular Probes, Fisher Scientific), a highly selective fluorogenic dye, as previously reported [55]. MitoSOX Red reagent permeates live cells, and rapidly and selectively targets mitochondria. At the mitochondrial level, it is rapidly oxidized by superoxide; the oxidation of MitoSOX reagent leads to red fluorescence. Cells were incubated with 3 µM MitoSOX Red in Hanks’s balanced salt solution (HBSS) at room temperature for 25 min in the dark, and washed with dye-free HBSS. The intensity of red fluorescence after excitation at 510 nm was acquired at a frequency of 0.25 Hz and evaluated as a measure of mitochondrial superoxide accumulation.

### 4.9. Statistical Analyses

Statistical analyses were performed using GraphPad Prism (version 8.0) software (GraphPadPrism Software, Inc. San Diego, California). Multiple analyses comparing to the vehicle control were carried out using one-way ANOVA followed by Dunnett’s test. A Student’s *t*-test was used to compare two groups (paclitaxel or KY-20-22 vs. paclitaxel + KY-20-22). For all experiments, *p* < 0.05 was considered significant. CompuSyn software was used to analyze the combination index of the combined action of KY-20-22 and paclitaxel. A synergistic action of KY-20-22 and paclitaxel was determined based on the Combination Index CI theorem of Chou–Talalay (additive effect CI = 1, synergism CI < 1, and antagonism CI > 1) [56].

## Figures and Tables

**Table 1:** Combination of KY-20-22 and paclitaxel on cell survival assay.

**MDA-MB-231**			
**KY-20-22 (µM)**	**Paclitaxel (nM)**	**Survival (%)**	**Combination Index**
0.5	5	70.35	2.195
0.5	10	44.50	0.879
1	5	60.48	1.85
1	10	37.63	0.678
5	5	35.48	0.569
5	10	26.8	0.365
**MDA-MB-468**			
**KY-20-22 (µM)**	**Paclitaxel (nM)**	**Survival (%)**	**Combination Index**
0.5	1	68.32	1.860
0.5	5	45.67	0.966
1	1	50.25	1.324
1	5	27.69	0.453
5	1	45.89	0.841
5	5	15.45	0.312
